# The Half-Yearly Abstract of the Medical Sciences

**Published:** 1846-03

**Authors:** 


*The Half-Yearly Abstract of the Medical Sciences.* Edited by W. H. Ranking, M. D., Cantab., Physician to the Suffolk General Hospital. New York: J. & H. G. Langley, for July and December, 1845, pp. 373.

This Journal contains a practical and analytical digest of all that occurs for the previous six months, in the different departments of medicine, and thereby furnishing much that is useful and desirable for every physician to be in possession of. We have before noticed this valuable publication, and would again commend it to the dental as well as medical practitioner.

